# A network frame offers a promising transdisciplinary tool for understanding complex health and health care system problems like suicide

**DOI:** 10.1073/pnas.2402194121

**Published:** 2024-08-13

**Authors:** Bernice A. Pescosolido

**Affiliations:** ^a^Department of Sociology, Indiana University, Bloomington, IN 47405; ^b^Irsay Institute for Sociomedical Sciences, Indiana University, Bloomington, IN 47405

**Keywords:** health, health care, complex systems, networks

## Abstract

As health and health care systems continue to face massive challenges from local to global well-being, understanding the processes that lead to improvement or deterioration in human health has embraced a broad range of forces from genes to national cultures. Despite the many efforts to deploy a common framework that captures diverse drivers at scale, the common missing element is the absence of a flexible mechanism that can guide research within and across levels. This hinders both the cumulation of knowledge and the development of a scientific foundation for multiplex interventions. However, studies across disciplines using a wide variety of methods and measures have converged on “connectedness” as crucial to understanding how factors operate in the health space. More formally, a focus on the critical role of the network structure and content of key elements and how they interact, rather than just on the elements themselves, offers both a generalized theory of active factors within levels and the potential to theorize interactions across levels. One critical contemporary health crisis, suicide, is deployed to illustrate the Network Embedded Symbiome Framework. The wide range of health and health care research where networks have been implicated supports its potential but also cautions against inevitable limits that will require creative theorizing and data harmonization to move forward.

## Significance

Our understanding of human health and health care now points to the key role of many diverse factors from genes to global cultures. Providing a common, inclusive, evidence-based, and theoretically organized network framework holds the potential to accelerate research and policy efforts to improve the health of individuals and societies.

According to the most prestigious health organizations in the world, we are currently facing a multitude of public health crises. These range from general concerns about growing disparities and documented reversals in life expectancy (1, 2) to increases in specific diseases such as hypertension (3, 4) and mental illness (5, 6). Further, regular pandemics are seen as inevitable (7) and comorbid effects on substance use disorders, depression, and unmet needs are expected to accompany them (8–10). The moniker of crisis has further broadened to embrace health problems that are contested, heavily social in nature, outside the purview of medicine traditionally defined, or that exacerbate existing health system strains [e.g., childhood obesity, (11); gun violence, (12); loneliness, (13); climate change, (14); provider burnout, (15); racism, (16)]. The breadth of this list signals a recognition that health rises and falls as part of a complex system of forces from genes to global structures. Ironically, this represents a return to the early view of influential biomedical scientists of the 19th-century who saw medicine as both a social and medical science (e.g., Rudolf Virchow in Germany; see ref. 17, Guðmundur Hannesson in Iceland; see ref. 18). Nowhere is this more evident than in the fairly recent biomedical focus on the “social determinants of health,” an agenda that stands as more than a century-long priority for the sociomedical sciences (19). As Kress et al. (20:13853) point out, facing the coming health challenges will require coupling “systematic social change and scientific progress.”

This impetus to contextual-level thinking is not new to health research, but its utility has been accelerated by the development of newer complex system approaches across the scientific landscape. For example, parallel to Nobel Laureate Elinor Ostrom’s (21:419) concept of social–ecological systems that address natural resource crises, human health systems are likewise “composed of multiple subsystems and internal variables within these subsystems at multiple levels” and which “are relatively separable but interact to produce outcomes…which in turn feed back to affect these subsystems and their components.” However, unlike conventional multilevel models that capture the levels of “social ecologies,” “helix to health,” “neurons to neighborhoods,” “base pairs to bedside,” “compound to clinic,” or “cells to society” (e.g., refs. 22–24), scientific research needs to go past Ostrom’s core systems and level variables. Simply “borrowing” variables from social science to add to genetic or other biomedical databases does little to integrate transdisciplinary insights and leads to problems from greater respondent burden to analytic power complications. Rather, to truly integrate multilevel and multidisciplinary insights requires identifying common underlying mechanisms that mark how factors work together or in opposition to impact health and health care. Without this, the effort to intervene runs the risk of missing the unintended consequences of tinkering with problems identified as “wicked.” That is, wicked problems are not amenable to linear thinking but are continually evolving, marked by a dynamic set of interlocking issues and constraints, and are unlikely to have definitive solutions (25). Historically, common examples of unintended consequences in health and health care include the side effects of drugs, the inability of efficacious randomized clinical interventions to translate into effective community-based care, the noted spillover effects of pandemic lockdowns triggering mental health and substance use disorders, and the failure of standardized medical tools and techniques to operate well across diverse gender, cultural, and generational groups who tend to be excluded from initial studies (26).

In medical and public health research and intervention, steps in this direction came from at least three sources. A focus on complex systems science broke through existing research and policy barriers, produced findings with demonstrated utility, and accelerated the impact of national policies across several areas including obesity and tobacco control (27:358). Others point to the “destructive clarity” that the COVID-19 pandemic brought to the recognition of symbiotic complexity in human health (20, 28). Finally, at the US NIH, “translation” took on three distinct meanings which fostered innovation—incorporating scientific insights across disciplinary silos (transdisciplinarity), moving from science to practice (implementation), and making scientific discovery available to the public (dissemination) (29). Each is key to scientific progress, clinical practice, and improved population health.

Yet, despite countless and marked advances, having a comprehensive understanding of why some individuals are healthy and others not, why some diseases affect one but not another group, or why some medical systems produce better outcomes than others have been complicated by the differing elements, language, methods, and analytic tools that accompanied the increasing specialization of the natural, physical, biomedical, and social sciences over hundreds of years. But simply laying out the levels of active elements, as they operate independently, reveals the shortfall of most contemporary multilevel, multidisciplinary approaches. When each discipline contributes its wisdom, only an additive cumulation of knowledge is possible. The next, crucial step requires the integration of disciplinary knowledge into a broader interactive transdisciplinary framework (30). Doing so requires that five essential criteria are met: 1) all contextual levels with documented impact are included, 2) an underlying mechanism connects levels, is dynamic, and allows for a way to consolidate and manage research questions, 3) an analytic language, familiar to the range of sciences, is available to facilitate synergy, 4) all methodological tools proven useful across the sciences are leveraged, and 5) findings offer a tangible way to improve population health, whether through direct medical intervention, public health prevention, or larger scale social and policy change (31).

The goal here is to offer one approach that targets how network structures, content, and mechanisms underlie both the origins of ill health and how solutions can be crafted for better health outcomes. While a network approach is unlikely to solve all or any problems in research and treatment (e.g., monogenic diseases), it may provide a way out of synthesizing how many etiological factors documented by disciplines operate and deliver better blueprints for translating research into action for complex disorders. For example, rather than seeing something inherent in a race or ethnic group that leads to poor health or higher levels of mortality, a social network approach looks to both the bonding (internal to the group) and bridging (outward reaching from the group) ties that shape or reinforce access to health behaviors, resources, and effective health services. The course of action is not to fix “them” but to fix the connections to resources. Similarly, if a brain-gut connection is implicated in obesity, diabetes, and heart disease, the answer lies not in fixing an organ (e.g., bariatric surgery) but in understanding and altering the direct and indirect network paths in a complex system [e.g., semaglutide, (32)].

## Explanation through Illustration: The CDC’s Strategic Direction for Suicide Prevention

Based on a long history of suicide research across the social and behavioral sciences, the US Centers for Disease Control issued a strategic plan based on the critical role of “connectedness.” Connectedness is defined, in that report, as “the degree to which an individual or group is socially close, interrelated, or shares resources with other individuals or groups. Further, connectedness occurs within and between multiple levels of the social ecology, that is between and among individuals, families, schools and other organizations, neighborhoods, cultural groups, and society as a whole” (33). A network theoretic framework not only centers on the issue of connectedness, but generalizes it away from two overly simple ideas—that connectedness is always good, and having no connectedness is the same as having no networks in operation. More importantly, a network frame matches five criteria necessary for a generalized framework outlined above. A network approach has been used in studying everything from protein folding at the molecular level to trade patterns across the cross-national landscape. Combining the social network approach from the social sciences, which dates back to the turn of the 20th-century (e.g., ref. 34; see ref. 35) with the more recent development of network science, primarily from physics near the turn of the 21st century (e.g., ref. 36), there is a basic and rich lexicon of terms, models, and methods. While there are some unique distinctions and some confusion of terms (e.g., the meaning of “multiplexity”), the scientific toolkit is nonetheless shared broadly and used widely (31, 37). Importantly, a network approach also provides a tangible way to intervene through connections among individuals (e.g., personal, health care contacts), organizations (e.g., shared personnel, contracts), and even genes (e.g., timing of gene expression in a pathway) in a way that manipulating categories (e.g., gender, perceived social support) does not.

The National Research Council and the Institute of Medicine (now the National Academy of Medicine) were among the first scientific organizations to suggest the potential of human relationships as part and parcel of an integrated health approach, specifying their role as active ingredients, basic building blocks, and mediators of environmental influence (38, 39). However, the link between connectedness and health finds much earlier roots in social sciences, particularly a nineteenth century sociological analysis of suicide (40), sociometry applied to treatment in juvenile justice systems in the 1930s (41), anthropological considerations of wellness in the 1960s (42), and psychosocial epidemiological theories of host resistance and social support in the 1970s (43, 44).

### A Basic Foundation: The Predictive Plane.

Drawing from these diverse sources, social network theory provides a fundamental base for developing and testing hypotheses. In particular, Durkheim’s (40) empirical analyses offered a theoretical starting point for connectedness that has been used in both epidemiological and health services research (24, 45, 46). This was not a network theory, since data limitations of the historical period allowed only for “categorical groups that vary in their relative contribution to the suicide rate” to proxy the “structures of social relations.” (47:502; emphasis added).

**Fig. 1.**
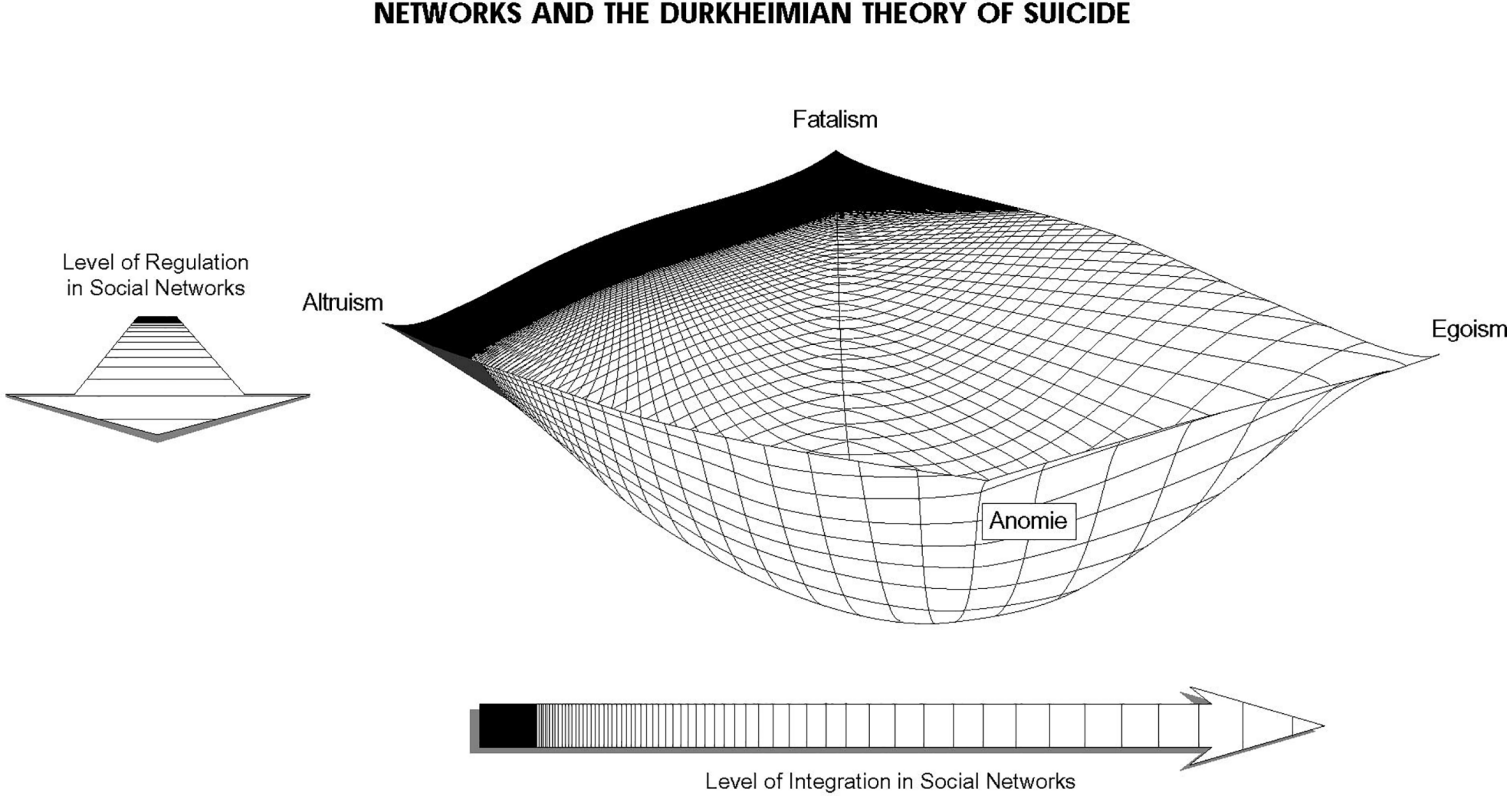
Theoretical predictive plane of network effects on health, disease, and health care. (Reprinted from ref. 46 with permission from Emerald Publishing Limited.)

The utility of this theoretical foundation comes from the basic predictive plane that Durkheim described and is graphically depicted in Fig. 1. Specifically, all social structures are defined by two key parameters of connection, integration, and regulation. When considered together, the potential structures exist on what resembles a social safety net with the optimal structural configuration lying at the nadir of the net where levels of both dimensions are moderate. This first “axis of variation” (48) running from right to left, is integration, indicative of ties that provide love, care, concern, and resource sharing. The second axis of variation running from front to back, is regulation, targeting norms that provide guidance, obligations, boundaries, behavioral sanctions, and barriers to resources. However, the key to this conceptualization is that neither axis of connectedness is inherently positive nor negative, though integration is often used to suggest facilitation (e.g., support), while regulation suggests suppression [e.g., coercion, (49)]. A more faithful interpretation suggests that surfeits and lacunae on any dimension are problematic, which has resonance with at least some findings in the biomedical sciences (see below). Poles indicate that extremes of “too much” or “too little” connectedness are toxic. Each pole produces a unique social structural type and, with it, a particularly high predisposition to suicide for different reasons. When social network ties are very sparse and loose, the social structure offers either too little integration (egoism, e.g., isolation) or too little regulation (anomie, e.g., suspension of regular norms) which are damaging to individuals in and of themselves but also are unable to cushion other traumas that individuals may face. Equally damaging to the individual, when there is too much integration (altruism, e.g., martyrs) or regulation (fatalism, e.g., cults), the structure of social ties resembles a hard and fragile wall, incapable of providing support or dampening the predisposition to suicidal behaviors. For example, on the internet, network ties to “extreme” communities (e.g., cults) or toxic alliances (e.g., suicide pacts) increase suicide (50). The optimal balance in social structure lies at the nadir of the net where both integration and regulation are in balance.

The power of this conceptualization is that it allows us to move away from the tendency to think of social and biological structures as fixed in time or as having only linear effects. Further, it allows for heterogeneous pathways to the same problematic health outcomes. That is, as a predictive plane, the safety net foundation of a multilevel health and health care model does not depict any one existing structure, but possible network structures derived from critical combinations of key elements at each level. Since neither elements nor the created structures are static but can move in any direction, the network conceptualization accommodates dynamic processes from plasticity to mutual adjustment to institutional social change. Further, while the graphic suggests that the two axes of variation are orthogonal, the number of axes and their relations to one another may be defined by particularities of considered case parameters. That is, the result is a unique theoretical plane, not easily described mathematically, where the interaction of case elements sets the bounds of the parameters which can vary dramatically across time, place, and level considered. This basic conceptualization has produced quite durable findings in suicide research, though not without contrary findings, concerns, adaptations, and alternatives (51, 52).

### A Fractal Approach to Multiple Levels: The Network Embedded Symbiome (NES) Framework.

**Fig. 2.**
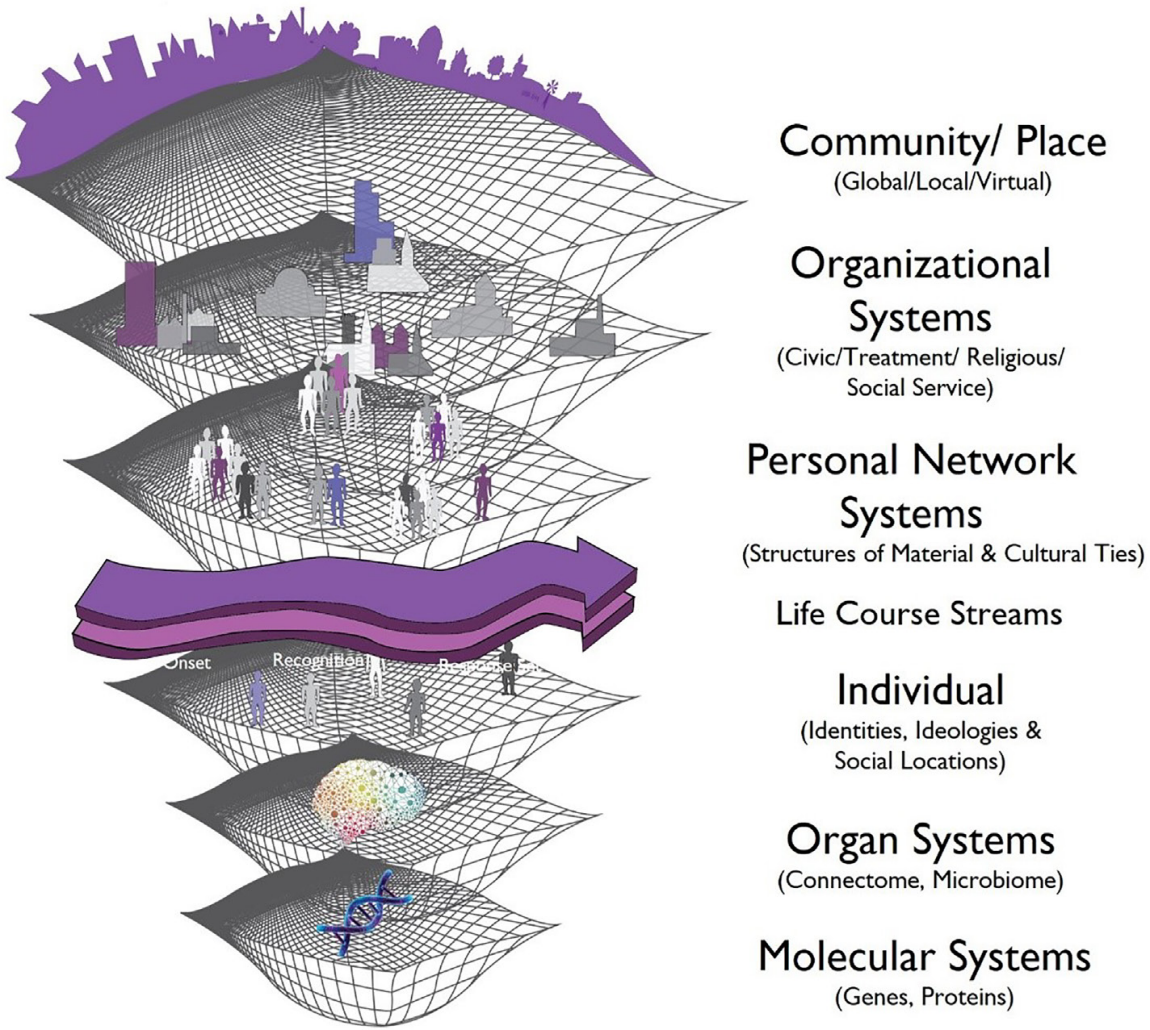
The NES framework for health and health care.

Deploying Abbott’s (53) translation of mathematical fractal imagery for social structures and processes, the idea of a “safety net” conceptualization produces the NES Framework [formerly the Social Symbiome, (37)] depicted in Fig. 2. While not exhaustive, these levels represent those most familiar to health researchers. Beginning with the interactive, contextual, and dynamic assumptions of complex systems science, the basic premise here is that social, technological, and organic systems are all dynamic, mutually contingent, and reinforcing or diluting. The convergence or clash among network systems drives the onset, recognition, response, and outcomes of health problems. In addition, it shifts the focus from elements (genes, neurons, individuals, nations) to interactions (influence, connections, transcription, cooperation, links) among elements, viewing network connections and what flows across them as the “engine of action.” For example, connectedness determines whether virtual social network communities represent supports for individuals for whom face-to-face social interaction is difficult or suicidogenic ones for others who encounter a barrage of negative, disconfirming messages (54). Similarly, social networks are likely to encourage, support, or even coerce their members into health care while denying need, discouraging, or even prohibiting service use for others (55, 56). At the organizational policy level, social networks influence whether comprehensive patient portals can be developed to produce better clinical outcomes (57) while, at the genetic level, multivariate connectivity of the sensorimotor putamen in human brain circuits is implicated in eating disorders (58).

In Fig. 2, the dark purple arrow represents dynamic health pathways [e.g., “illness careers” or disease course; (45)], with any number of basic but not predetermined linear stages (e.g., onset, recognition, health care response, outcomes). The lighter purple arrow represents an explicit focus on time, key turning points, biographical disruptions, and trends (53, 59). For example, political scientists have suggested that election years represent times of greater sociopolitical integration, generally resulting in lower suicide rates compared to nonelection years (e.g., ref. 60). In the most crucial sense, however, the potential of the fractal structure highlights biological and social embeddedness. The former, biological embedding (61, 62) targets the process whereby social factors get “under the skin” or “into the mind” while the latter, social embedding (adapted from ref. 63) references the process whereby biological factors support, enhance, limit, or destroy the capacity for social interactions (29).

## Resonance of the NES Frame with Existing Suicide Research across Levels

Suicide represents one global crisis targeted by the WHO, the NIH, and NASEM. Long associated with issues of human social relations, including loneliness in the recent Surgeon General’s Report (13), and with depression as the leading cause of disability in the Global Burden of Disease studies (e.g., ref. 64), suicide has been researched from many perspectives—psychiatric, psychological, biological, economic, sociological among others. Over many years, calls for understanding the complexity and multiple factors involved provide an evidential basis for incorporating elements into a network perspective.

### Personal Network Systems.

This level represents the most direct application of the network theory of suicide since individuals’ ties form the elemental focus. These personal networks represent the most common and direct application of a network perspective in health. Generally, social networks have been shown to buffer stress, transmit norms, affect the adoption of innovation, and be a potent source for behavioral change and the uptake of treatment services as well as vector of health risk, morbidity, and mortality (e.g., refs. 37 and 65–69). Relevant to suicide, research suggests a fairly consistent alignment with the network theory of suicide where both no ties, few ties, or broken ties, and ties with extreme groups or contagion, are associated with a greater predisposition to suicide (51, 52). As Cero et al. (70) summarize, individuals with elevated suicide risk are both disproportionately connected with one another and less connected to their wider networks. Importantly, even their simple network intervention decreased suicide risk without harming healthy individuals.

**Fig. 3.**
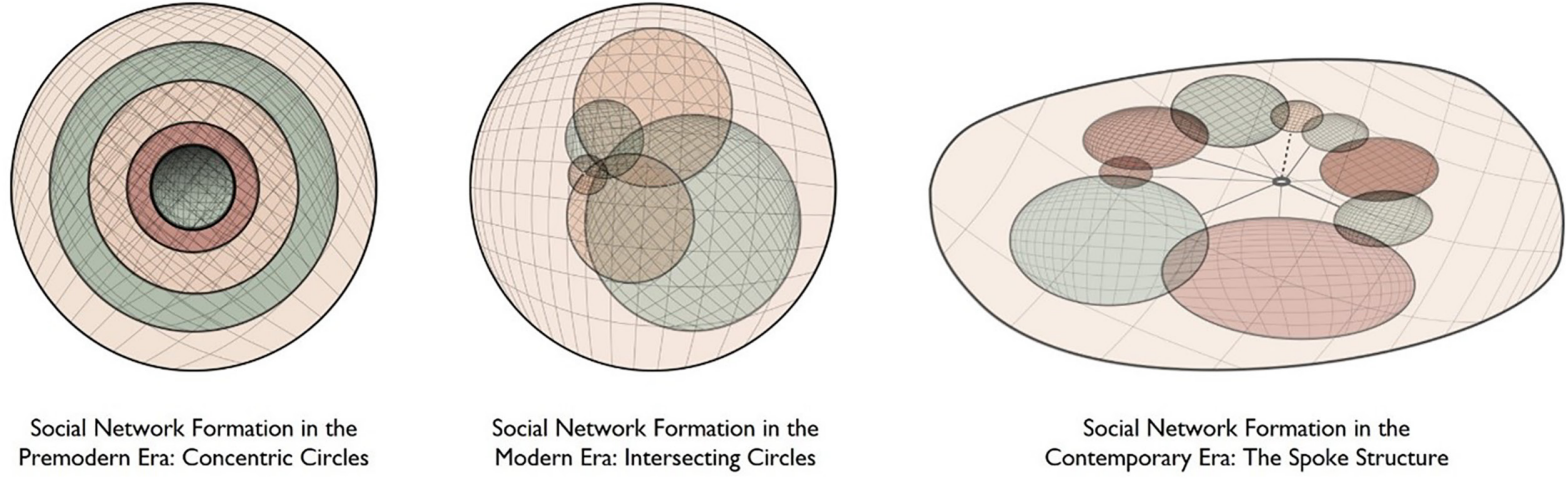
Network archetypes in historical and potentially individual contexts.

However, this level has greater potential. Drawing from Wellman and Gulia’s (71) insight that social networks are more than collections of tie characteristics, research has yet to take advantage of whether and how the overall structure of individuals’ networks place individuals at different places on the predictive plane. For example, Simmel’s (34) original two forms of “sociation” depicted premodern, agrarian (concentric) and modern, industrial social structures (intersecting), with a third postmodern (spoke) structure added more recently, were reconsidered as possible structures for personal ties in line with social determinants of health (Fig. 3). Rather than only as historical archetypes, Pescosolido and Rubin (72) suggested that such types have existed in all societies. For example, the concentric circle form (Fig. 3, left image) aligns with William Julius Wilson’ (73) conclusion that the true disadvantage of urban-dwelling African Americans lies in the concentration of homogenous socioeconomic ties cutoff from employment as industrial work moved to the suburbs. The configuration of strong bonding but weak bridging ties is implicated both in the mental health paradox (74) and by implication in the historically lower suicide rate among African-Americans. More recently, a review of trends and precipitating circumstances suggests network-based interventions may be most promising to counter the rise in suicide among black youth, particularly boys (75).

### Individual Networked Systems.

Three major issues at this level are central to the NES Framework. First, social characteristics are proxies for social relationships. By their very nonrandom nature, sociodemographics tap into the presence, absence, or change in the likelihood of having, gaining, or losing ties. Since the beginning of suicide research, individuals who live in one-person households, have no or low connections to institutions like religion or work, report discordant social relationships, or hold status that suggest broken ones [e.g., divorce, widowed, (51, 76, 77)] have higher suicide risk. On the individual level, the alignment of a network theory with a current predominant theory of suicide (78) translates integration (i.e., thwarted belongingness) and regulation (i.e., perceived burdensome) into individual psychological expression. In some cases, research targets (e.g., completed suicide) may only have access to sociodemographic characteristics but creative data harmonization can bridge the gap. For example, a longstanding concern with the influence of religion on suicide may proxy the tendency of some religious groups to foster ties only with others in the same religious group (45). While increasing ties that provide support (bonding capital), lowers the risk of suicide, the occurrence of a crisis (e.g., a threat from outside the group) in these networks can shift the network balance and move the networks close to the toxic poles in Fig. 1.

Second, the continued utility of sociodemographics may lie in their important interactive effects across levels since intervention strategies should “extend beyond the individual” and yet be tailored to the “individual-environment constellation” (79). On the one hand, among emerging adults, females and males are likely to activate social ties for different reasons including helping them gain self-control over problems or achieve awareness of the problem, respectively (80). But Hinshaw and Kranz’s (81) prediction that the “triple bind” in high schools has a greater impact on high school girls is now reflected in the gender disparity in rising youth suicide rates. On the other hand, web communication appears to be more of a risk factor for suicide for boys than for girls (54). Finally, other “groups” are more likely to have social networks characterized by substance abuse, the loss of social networks, or social isolation, each elevating suicide risk [e.g., American Indian or homeless youth, (79, 82); Native Hawaiians, Black Caribbean youth, young Black African-American men; Finnish youth in Sweden, (77, 83), respectively].

Third, sociodemographic effects can also interact with individuals’ psychological identity, evaluations, and emotions. That is, the formation of “the self” is a process that fundamentally involves others in an interactive linkage between internal processes of self-verification, identity, and social structures (84). For example, at risk youth overestimate their friends’ suicidal behaviors which, in turn, exacerbates their own risk (85).

### Organizational Networks.

Networks at this level have four targets and include “clients” (e.g., students, patients), “staff” (e.g., teachers, physicians), and their interactive connections [student-teacher relationship, physician–patient interaction, (86)]. First, organizations facilitate or stifle the development of network ties between and among clients and staff. Both the lack of “good” and the presence of “bad” social ties as well as the tendency to form homophilous relationships have been implicated in suicide clusters in affected schools and businesses (87). Second, organizations provide cultural scripts for behavior and create a cultural climate. As Abrutyn et al. (88:113) documented in a high school experiencing problems, suicide was “more easily imagined by youth because the community collectively constructed a widely shared local cultural script for suicide.” Third, organizations routinely provide or fail to provide relevant services. Attention to school mental health has increased dramatically in recent years, with school systems deciding whether to integrate suicide prevention programs (89). Fourth and finally, interorganizational networks create “systems,” helping to explain if, where and why individuals are likely to “fall through the cracks” because of insufficient coordination, training, or referrals (90). Not limited to health care, connectedness between and among community organizations matter with, for example, police institutions in some countries controlling most suicide information and, as a result, a community’s ability to provide a coordinated response (91).

### Geographically Based Network Systems.

Much suicide research relies on area-based rates, documenting clear and interesting effects, though not without inconsistencies. For example, the male suicide rate tends to be higher across counties and countries with some exceptions [e.g., Uzbekistan, Southeast Asia, (92)], and in places characterized by higher poverty (93), economic crises (94), or fractured families [particularly divorce rates, (95)]. Countries with a high level of religious heterogeneity tend to have higher suicide rates (96). Within the United States, the greater presence of different denominations (e.g., mainline protestant) are associated with higher county group suicide rates (45). Cross-national analyses, harmonizing data from the European Social Survey and suicide rate data, reveal community social support associated with lower suicide rates for both males and females, though stronger impact on the latter (97).

Place-based suicide rate data often have been used and interpreted not for the effect of place but rather as a proxy for individual-level risk factors, despite the ecological fallacy. However, urban geographers’ long-term concern with networks of place has produced a frame that mirrors the complexity of the top level in Fig. 2, including cities as networks and networks of cities (98). Importantly, conceptualizing the relevant level of geographic analyses is far from understood. For example, the geography of suicide among elderly residents of Hong Kong failed to reflect the common rural-urban hypothesis while smaller area suicide clusters appeared to be associated with better services and resources (99). Geography, in and of itself, is critical for understanding the development, tailoring, and placement of care and prevention in suicide.

### Biological Networked Systems.

The resurgence of systems biology, targeting human cellular-level networks, includes a shift to signaling and transport links, for example, with brain function relying on efficient communication between distinct brain systems. “Hubs of dysfunction” (100), “rich club organization” (101), and “connectome-based functional connectivity” (102) have been explored as markers of suicide attempts (also ref. 103). Similarly, a network analysis found community clustering for those with major depression, but also among those with suicidal ideation, a more complex difference that destroyed betweeness-centrality in key network structures (101). In an interesting, novel focus, Chin Fatt et al. (104) identified a gut microbial co-occurrence network significantly associated with clinical anxiety, a documented suicide risk factor, in a cohort of individuals with depression. Here, the network approach is still relatively new and focused on suicidal ideation or attempts since data are collected from live subjects.

### Molecular Networked Systems.

Despite the long history of family, twin and adoption studies that suggest possibilities, evidence for the genetics of suicide continues to be unclear. Statistically significant markers for suicide (i.e., genome-wide significant single nucleotide polymorphisms) have not been located, but GWAS approaches using polygenic risk score analysis suggest a polygenic architecture, and a genomic structural equation model identified 98 subnetwork hub genes associated with a “common factor” for suicidal behavior (105). This reflects the shift from gene-centric to network-centric approaches with some work focusing on eRNAs (non-protein-coding) function as endogenous network control molecules facilitating gene-to-gene communication and multitasking (106). Although not directly related to suicide, two highly resonant findings that support the “safety net” conceptualization can be found in copy number variant (CNV) research. First, the Smith–Magenis syndrome (SMS) and Potocki–Lupski syndrome (PTLS) are reciprocal contiguous gene syndromes (17p11.2 region) where failure to replicate gene segments (“too little” microdeletion) results in the former while adding extra copies (“too much” microduplication) results in the later. As with suicide types on either axis of variation described earlier, these behavioral syndromes share the same genomic region but result in distinct “clinical manifestations and behavioral issues” which “mirror traits…on opposite ends of a given phenotypic spectrum” (107:159). Second, a recent study on a rare liver cancer, fibrolamellar carcinoma (FLC) suggested that an overproduction of the PKA protein, rather than the commonly accepted origin theory (i.e., a fusion protein) is implicated in the cause (108). Interestingly, press coverage referred to the finding as “a lack of inhibition” (109), similar to the regulation axis of the safety net framework.

## Why an Additive Approach Is Insufficient: Early Evidence on Cross-Level Interconnections

Networks operating at one level can counter ties at another or reinforce their impact. Because individuals may have overregulative networks at one level (e.g., cults which require cutting outside ties), supportive networks at other levels (preexisting family and friend networks) may counter suicide risk. Conversely, having experiences of abuse or trauma within the family network may potentially trigger gene-based suicide risk (i.e., biological embedding). Sokolowski et al.’s (110) research suggests multiple cross-level effects between genetic profiles, trauma, and suicidal behavior. Few studies have pursued this agenda, but some research is suggestive.

### Suicide and “Sameness”.

Guided by the network concepts of selective attachment or homophily, we (111) examined individual and aggregate level data on completed suicide, testing the hypothesis that an individual’s risk factor may be lessened if they live in an area with “like-others” and magnified if they are isolated in confronting life challenges. Using 5 y of harmonized data from the National Violent Death Reporting System and the American Community Survey, cross-level demographic similarity or “sameness” mitigated the individual risk of suicide from known stressors (e.g., divorce, unemployment) but for some groups that have experienced historical trauma (American Indians, Alaska Natives), sameness aggravated the risk of suicide. Here, the individual risk posed by known risk factors that can sever critical ties (e.g., unemployment) can be mitigated by the observation of a similar fate for others (e.g., loss of income and ties) that shifts the blame to the system rather than the individual.

### Suicide, Regions, and Religious Presence.

As described earlier, the effect of the population affiliated with different US denominations is associated with different suicide rates in accordance with the likelihood of network religious closure in personal networks (45). However, religions not only facilitate personal ties but create organizational support networks in their communities (e.g., Catholic Charities). Under a multilevel network theory, suicide may be lower for different religious groups in their areas of historical concentration. An analysis of regional effects on the religion-suicide link revealed clear support—for example, while the effect of Judaism is protective and small overall, it is large in the Northeast and reversed in the South (112).

### Suicide, Seasons, Historical Time, and Social Activity.

From the earliest studies onward, suicide in the Global North has been highest in the spring and summer, reversing in the winter, and with a “December trough” [e.g., drop of 39% for men, 20% for women in Switzerland, (113)]. While some attribute this to temperature or other environmental effects, others suggest the increase in social activity in the warmer times and the bonding potential of a heavy rotation of ritual, holiday season events in the colder times reflect social integration change. Moreover, given secular trends, a “new move” in research argues that the seasonality effect is diminishing and may eventually disappear, even as seasonal climate differences are intensifying (114).

We are only at the early stages of exploring the complexity of connectedness across levels of biological and social systems. Research adopting a network approach has used virtually every data type in the scientific toolbox from genetic assays, imaging, surveys, interviews, online signals, ethnography, to archives, both within and across levels of analysis. But network data are often more difficult to collect and have innate limits in some cases [e.g., suicide, (47, 111)]. Here, the willingness to use creative solutions where the best is not the enemy of the good may present the only path forward. Flexibility in approach is essential. The NES suggests the fractal structure across levels with decent preliminary evidence in the social sphere and some suggestive data in the biological sphere. However, exactly what the axes of variation or the key elements are across levels, or that it is even a viable conceptualization has yet to be considered.

This leads us to ask many questions that require creative integration in theorizing and harmonizing the rich and deep stocks of knowledge currently held by the wide range of fields that seek to improve health and the human condition. The essential element for success in pushing this agenda forward is, not surprisingly, the creation of multidisciplinary network teams with differential network expertise that share the willingness to collaborate given the centrality of connectedness. Acknowledging the power and status hierarchy or “pecking order” in the sciences, this is more easily established in theory than it is carried out in practice. Social science has established that stratification is omnipresent and research groups that require leadership are unlikely pioneers in establishing equality. Even among partners anxious to break new ground together, limits of time, money, data sources, etc., will confront issues of whose tools or ideas are more powerful and deserving of those limited resources. However, we are well past the point in deploying a network approach where the strengths and limits of the biomedical, natural, and social sciences are not acknowledged. Medicine’s traditional focus on “the disease on the body in the bed,” natural science’s relative lack of concern about missing data, and social science’s only nascent ability to handle big data are just a few of the issues that require a deeper sense of commitment to a larger enterprise than to the tendency to fall back on standard ideas and practices. Finally, just as there are phenomena that may not be a good fit for a network approach, there will also be scientists for whom this is not the best fit. Waiting until a problem arises, and hoping they will not, is poor practice while understanding, discussing, and setting the foundations from the beginning in creating teams to break through existing scientific biases is essential.

## Conclusion

The NES Framework provides a network science variant of complex systems science that integrates past insights into network effects in health and health care, provides a common analytic language, and offers a broad-based toolbox of methods and analytic approaches. The NES goes beyond juxtaposition to the interpenetration of disciplinary epistemologies as Mazzocchi (30) suggests. A model predictive plane is based on a translation of a classic protonetwork theory of gaps and excesses in integration and regulation combined with fractal imagery across ecological levels that emphasizes both social and biological embedding. Drawing from over 100 y of attention in the social sciences to only a decade in genetics, the existing base of research, both for understanding suicide and other health and health care issues, is promising.

Confronting scientific limits in health and health care through a common lens provides a way to break through the efficacy-effectiveness barrier, especially for suicide where contextual or multiplier effects have rarely been considered (115). Understanding the structure and function of network ties, the “wires” across which influences flow, offers more direct intervention targets. Mapping the structure of a social network that produces loneliness, for example, not only helps us unravel suicide risk, but provides a clear target for change that assessing individuals’ perception of their social support or report of their loneliness does not.

Even more importantly, a network perspective provides a more direct next step to reduce health disparities. The social determinants of health agenda leveraged sociodemographic categories (e.g., social class, gender, race/ethnicity) to mark differential risk, unequal care, and critical differences in outcomes. While requiring continued special attention to categories, the common underlying issue across the social determinants of health is inequality, which sociologist Charles Tilly (116) called “durable” because it operates at the mesolevel, between macrostructures and individual characteristics, where networks exist. He argues that where both individual or macrostructures are either difficult or impossible to change, social networks represent the mechanisms that produce, maintain, and amplify cumulative disadvantage. They are mutable.

In other words, categories such as race or social class are just gross shorthands for structures of social relationships which translate to indirect factors to target changes that can improve population health. Moving more directly to networks in research and even in treatment (e.g., ref. 117) aligns with the medicine’s move toward precision or tailored medicine, broadly defined. Taking this direction does not eliminate the focus of social characteristics in research’ rather, as the NES Framework suggests, understanding the interaction of network structures and sociodemographic characteristics represents a powerful direction.

In the end, the fundamental basis of this approach, and the foundation of the NES Framework, is human connectedness. But connectedness may be more universal. The power of social relationships on life chances appears to be conserved across mammals with resonance in other species. Specifically, a recent analysis of almost 1,000 mammal species using a crude trichotomy of solitary, pair-based, and communal living, revealed that “group-living species generally live longer than solitary species, and that the transition rate from a short-lived state to a long-lived state is higher in group-living than non-group-living species, altogether supporting the correlated evolution of social organization and longevity” (118). Further, eastern garter snakes display not only sociability but selective attachment (116), and, among Southern Pacific rattlesnakes, “companions” can reduce their biological responses to stress, displaying the calming effects associated with social buffering (119). The potential for linking connectedness across the range of problems affecting human health, including resource systems and climate effects, is striking.

Returning to the wisdom of Ostrom’s work (21), we acknowledge that the biomedical and social sciences have developed independently, do not easily combine, and do not meet at the table with an equal social status. Further, the NES provides only one recommendation to push forward; and as Ostrom (21) reminds us, one-size-fits-all recommendations frequently fail. But the signs are encouraging.

## Data Availability

There are no data underlying this work.

## References

[1] M. Marmot, Health equity in England: The Marmot review 10 years on. BMJ **368**, m693 (2020). 32094110 10.1136/bmj.m693

[2] A. S. Venkataramani, R. O’Brien, A. C. Tsai, Declining life expectancy in the United States: The need for social policy as health policy. JAMA **325**, 621–622 (2021). 33591352 10.1001/jama.2020.26339

[3] P. A. Berek, D. Irawati, A. Y. S. Hamid, Hypertension: A global health crisis. Ann. Clin. Hypertension **5**, 008–011 (2021).

[4] World Health Organization, A Global Brief on Hypertension: Silent Killer, Global Public Health Crisis (WHO, Geneva, Switzerland, 2013).

[5] G. Thornicroft , The lancet commission on ending stigma and discrimination in mental health. Lancet **400**, 1438–1480 (2022). 36223799 10.1016/S0140-6736(22)01470-2

[6] World Health Organization, World Mental Health Report: Transforming Mental Health for All (World Health Organization, Geneva, Switzerland, 2022), p. 272.

[7] D. M. Hartley, E. N. Perencevich, Public health interventions for COVID-19: Emerging evidence and implications for an evolving public health crisis. JAMA **323**, 1908–1909 (2020). 32275299 10.1001/jama.2020.5910

[8] J. M. Clay, M. O. Parker, Alcohol use and misuse during the COVID-19 pandemic: A potential public health crisis? Lancet Public Health **5**, e259 (2020). 32277874 10.1016/S2468-2667(20)30088-8 PMC7195126

[9] T. A. Schwetz, T. Calder, E. Rosenthal, S. Kattakuzhy, A. S. Fauci, Opioids and infectious diseases: A converging public health crisis. J. Infect. Dis. **220**, 346–349 (2019). 30941402 10.1093/infdis/jiz133 PMC6941614

[10] K. C. Sahoo , Challenges in maternal and child health services delivery and access during pandemics or public health disasters in low-and middle-income countries: A systematic review. Healthcare (Basel) **9**, 828 (2021). 34209238 10.3390/healthcare9070828 PMC8306470

[11] C. B. Ebbeling, D. B. Pawlak, D. S. Ludwig, Childhood obesity: Public-health crisis, common sense cure. Lancet **360**, 473–482 (2002). 12241736 10.1016/S0140-6736(02)09678-2

[12] H. Bauchner , Death by gun violence—A public health crisis. JAMA **318**, 1763–1764 (2017). 29052721 10.1001/jama.2017.16446

[13] Office of the Surgeon General, “Publications and reports of the surgeon general” in Our Epidemic of Loneliness and Isolation: The U.S. Surgeon General’s Advisory on the Healing Effects of Social Connection and Community (US Department of Health and Human Services, Washington, DC, 2023). 37792968

[14] A. R. Hwong , Climate change and mental health: Implications for the psychiatric workforce. Psychiatr. Serv. **73**, 592–595 (2022). 34369808 10.1176/appi.ps.202100227

[15] V. H. Murthy, Confronting health worker burnout and well-being. N. Engl. J. Med. **387**, 577–579 (2022). 35830683 10.1056/NEJMp2207252

[16] D. Devakumar , Racism, the public health crisis we can no longer ignore. Lancet **395**, e112–e113 (2020). 32534630 10.1016/S0140-6736(20)31371-4 PMC7289562

[17] T. M. Brown, E. Fee, Rudolf Carl Virchow: Medical scientist, social reformer, role model. Am. J. Public Health **96**, 2104–2105 (2006). 17077410 10.2105/AJPH.2005.078436 PMC1698150

[18] S. Nannini, From reception to invention: The arrival of concrete to Iceland and the rhetoric of Guðmundur Hannesson. Arts **7**, 68 (2018).

[19] World Health Organization, “Social determinants of health” (Tech. Rep. SEA-HE-190, WHO Regional Office for South-East Asia, New Delhi, 2008).

[20] W. J. Kress, J. A. K. Mazet, P. D. N. Hebert, Intercepting pandemics through genomics. Proc. Natl. Acad. Sci. U.S.A. **117**, 13852–13855 (2020). 32493752 10.1073/pnas.2009508117 PMC7322079

[21] E. Ostrom, A general framework for analyzing sustainability of social-ecological systems. Science **325**, 419–422 (2009). 19628857 10.1126/science.1172133

[22] U. Bronfenbrenner, The Ecology of Human Development: Experiments by Nature and Design (Harvard University Press, Cambridge, MA, 1979).

[23] National Institutes of Health, Office of Behavioral and Social Sciences Research, *Progress and Promise in Research on Social and Cultural Dimensions of Health: A Research Agenda* (Office of Behavioral and Social Sciences Research, 2001).

[24] Institute of Medicine Committee on Pathophysiology Prevention of Adolescent & Adult Suicide, Reducing Suicide: A National Imperative, S. K. Goldsmith, T. C. Pellmar, A. M. Kleinman, J. Bunney, E. William, Eds. (National Academies Press, Washington, DC, 2002), 10.17226/10398. 25057611

[25] H. W. J. Rittel, M. M. Webber, Dilemmas in a general theory of planning. Policy Sci. **4**, 155–169 (1973).

[26] E. Burches, M Burches, Efficacy, effectiveness and efficiency in the health care: The need for an agreement to clarify its meaning. Int. Arch. Public Health Commun. Med. **4**, 035 (2020).

[27] D. A. Luke, K. A. Stamatakis, Systems science methods in public health: Dynamics, networks, and agents. Ann. Rev. Public Health **33**, 357–376 (2012). 22224885 10.1146/annurev-publhealth-031210-101222 PMC3644212

[28] E. A. Zerhouni, Translational and clinical science–Time for a new vision. N. Engl. J. Med. **353**, 1621–1623 (2005). 16221788 10.1056/NEJMsb053723

[29] B. A. Pescosolido , “The social symbiome framework: Linking genes-to-global cultures in public health using network science” in Handbook of Applied System Science, Z. Neal, Ed. (Routledge, Abingdon, 2016), pp. 25–48, 10.4324/9781315748771.

[30] F. Mazzocchi, Scientific research across and beyond disciplines: Challenges and opportunities of interdisciplinarity. EMBO Rep. **20**, e47682 (2019). 31040110 10.15252/embr.201947682 PMC6549017

[31] B. A. Pescosolido, “Organizing the sociological landscape for the next decades of health and health care research: The network episode model III-R as cartographic subfield guide” in The Handbook of the Sociology of Health, Illness, and Healing: Blueprint for the 21st Century, B. A. Pescosolido, J. K. Martin, J. D. McLeod, A. Rogers, Eds. (Springer, New York, 2011), pp. 39–66.

[32] E. Roh, K. M. Choi, Hormonal gut-brain signaling for the treatment of obesity. Int. J. Mol. Sci. **24**, 3384 (2023). 36834794 10.3390/ijms24043384 PMC9959457

[33] National Center for Injury Prevention and Control (US), Connectedness as a strategic direction for the prevention of suicidal behavior. https://stacks.cdc.gov/view/cdc/11796. Accessed 7 July 2023.

[34] G. Simmel, Conflict and the Web of Group Affiliations (Free Press, New York, 1955).

[35] L. C. Freeman, The Development of Social Network Analysis: A Study in the Sociology of Science (Empirical Press, Vancouver, BC, 2004), p. 205.

[36] A. Vespignani, Twenty years of network science. Nature **558**, 528–529 (2018). 29941900 10.1038/d41586-018-05444-y

[37] B. A. Pescosolido, Of pride and prejudice: The role of sociology and social networks in integrating the health sciences. J. Health Soc. Behav. **47**, 189–208 (2006). 17066772 10.1177/002214650604700301

[38] J. P. Shonkoff, D. A. Phillips, From Neurons to Neighborhoods: The Science of Early Childhood Development (Institute of Medicine Committee Report National Academy Press, Washington DC, 2000). 25077268

[39] B. Singer, C. Ryff, New Horizons in Health: An Integrative Approach (National Academy Press, Washington DC, 2001). 20669490

[40] E. Durkheim, Suicide (Free Press, New York, 1951 [1897]).

[41] J. L. Moreno, Sociometry in relation to other social sciences. Sociometry **1**, 206–219 (1937).

[42] E. Bott, Family and Social Network (Tavistock Press, London, 1957).

[43] D. A. Luke, J. K. Harris, Network analysis in public health: History, methods, and applications. Ann. Rev. Public Health **28**, 69–93 (2007). 17222078 10.1146/annurev.publhealth.28.021406.144132

[44] L. F. Berkman, S. L. Syme, Social networks, host resistance, and mortality: A nine-year follow-up study of Alameda County residents. Am. J. Epidemiol. **109**, 186–204 (1979). 425958 10.1093/oxfordjournals.aje.a112674

[45] B. A. Pescosolido, S. Georgianna, Durkheim, religion, and suicide: Toward a network theory of suicide. Am. Sociol. Rev. **54**, 33–48 (1989). 11616426

[46] B. A. Pescosolido, J. A. Levy, The role of social networks in health, illness, disease and healing: The accepting present, the forgotten past, and the dangerous potential for a complacent future. Soc. Netw. Health **8**, 3–25 (2002).

[47] P. S. Bearman, The social structure of suicide. Sociol. Forum **6**, 501–524 (1991).

[48] K. Erikson, Everything in Its Path: Destruction of Community in the Buffalo Creek Flood (Simon and Schuster, New York, 1976).

[49] G. Huitsing , Univariate and multivariate models of positive and negative networks: Liking, disliking, and bully-victim relationships. Soc. Netw. **34**, 645–657 (2012).

[50] K. Mok, A. F. Jorm, J. Pirkis, Suicide-related Internet use: A review. Aust. N. Z. J. Psychiatry **49**, 697–705 (2015). 25698810 10.1177/0004867415569797

[51] M. Wray, C. Colen, B. A. Pescosolido, The sociology of suicide. Ann. Rev. Sociol. **37**, 505–528 (2011).

[52] A. S. Mueller, S. Abrutyn, B. Pescosolido, S. Diefendorf, The social roots of suicide: Theorizing how the external social world matters to suicide and suicide prevention. Front. Psychol. **12**, 621569 (2021). 33868089 10.3389/fpsyg.2021.621569 PMC8044307

[53] A. Abbott, Chaos of Disciplines (University of Chicago Press, Chicago, 2001).

[54] F. Y. Tseng, H. J. Yang, Internet use and web communication networks, sources of social support, and forms of suicidal and nonsuicidal self-injury among adolescents: Different patterns between genders. Suicide Life Threat Behav. **45**, 178–191 (2015). 25255896 10.1111/sltb.12124

[55] B. A. Pescosolido, H. D. Green Jr., Who has mental health problems? Comparing individual, social and psychiatric constructions of mental health. Soc. Psychiatry Psychiatr. Epidemiol. **59**, 443–453 (2024). 37069339 10.1007/s00127-023-02474-4 PMC10108793

[56] H. D. Green Jr., B. A. Pescosolido, Social pathways to care: How community-based network ties shape the health care response of individuals with mental health problems. Soc. Psychiatry Psychiatr. Epidemiol. **59**, 431–442 (2024). 37072564 10.1007/s00127-023-02476-2 PMC10113125

[57] T. Otte-Trojel, T. G. Rundall, A. de Bont, J. van de Klundert, Can relational coordination help inter-organizational networks overcome challenges to coordination in patient portals? Int. J. Healthcare Management **10**, 75–83 (2017).

[58] A. R. Wang , Human habit neural circuitry may be perturbed in eating disorders. Sci. Transl. Med. **15**, eabo4919 (2023). 36989377 10.1126/scitranslmed.abo4919

[59] B. L. Perry, B. A. Pescosolido, Social network dynamics in the face of biographical disruption: The case of “first timers” with mental illness. Am. J. Sociol. **18**, 134–175 (2012).

[60] M. Boor, Relationships between unemployment rates and suicide rates in eight countries, 1962–1976. Psychol. Rep. **47**, 1095–1101 (1980). 7220739 10.2466/pr0.1980.47.3f.1095

[61] C. Hertzman, The biological embedding of early experience and its effects on health in adulthood. Ann. N. Y. Acad. Sci. **896**, 85–95 (1999). 10681890 10.1111/j.1749-6632.1999.tb08107.x

[62] S. Toyokawa, M. Uddin, K. C. Koenen, S. Galea, How does the social environment “get into the mind”? Epigenetics at the intersection of social and psychiatric epidemiology. Soc. Sci. Med. **74**, 67–74 (2012). 22119520 10.1016/j.socscimed.2011.09.036 PMC3246041

[63] M. Granovetter, Economic action and social structure: The problem of embeddedness. Am. J. Sociol. **91**, 481–510 (1985).

[64] T. Vos , Global burden of 369 diseases and injuries in 204 countries and territories, 1990–2019: A systematic analysis for the Global Burden of Disease Study 2019. Lancet **396**, 1204–1222 (2020). 33069326 10.1016/S0140-6736(20)30925-9 PMC7567026

[65] K. de la Haye, H. D. Green Jr., D. P. Kennedy, M. S. Pollard, J. S. Tucker, Selection and influence mechanisms associated with Marijuana initiation and use in adolescent friendship networks. J. Res. Adolesc. **23**, 474–486 (2013). 10.1111/jora.12018 PMC3811150 24187477

[66] L. M. Koehly , A social network analysis of communication about hereditary nonpolyposis colorectal cancer genetic testing and family functioning. Cancer Epidemiol. Biomarkers Prev. **12**, 304–313 (2003). 12692104

[67] B. A. Pescosolido, “Illness careers and network ties: A conceptual model of utilization and compliance” in Advances in Medical Sociology, G. L. Albrecht, J. A. Levy, Eds. (JAI Press, CT, 1991), pp. 161–184.

[68] B. A. Pescosolido, Beyond rational choice: The social dynamics of how people seek help. Am. J. Sociol. **97**, 1096–1138 (1992).

[69] T. W. Valente, Social Networks and Health: Models, Methods, and Applications (Oxford University Press, Oxford, 2010), p. 296.

[70] I. Cero, M. De Choudhury, P. A. Wyman, Social network structure as a suicide prevention target. Soc. Psychiatry Psychiatr. Epidemiol. **59**, 555–564 (2024). 37344654 10.1007/s00127-023-02521-0

[71] B. Wellman, M. Gulia, “The network basis of social support: A network is more than the sum of its ties” in Networks in the Global Village, B. Wellman, Ed. (Westview Press, Boulder, CO, 1999), pp. 83–118.

[72] B. A. Pescosolido, B. A. Rubin, The web of group affiliations revisited: Social life, postmodernism, and sociology. Am. Sociol. Rev. **65**, 52–76 (2000).

[73] W. J. Wilson, The Truly Disadvantaged: The Inner-City, the Underclass, and Public Policy (University of Chicago Press, Chicago, 1990).

[74] P. Smith, P. Nicaise, V. Lorant, Social integration of people with non-psychotic mental illness over the last 2 decades: The widening gap in the adult population in Belgium. Soc. Psychiatry Psychiatr. Epidemiol. **58**, 723–733 (2023). 35606460 10.1007/s00127-022-02302-1

[75] A. H. Sheftall , Black youth suicide: Investigation of current trends and precipitating circumstances. J. Am. Acad. Child Adolesc. Psychiatry **61**, 662–675 (2022). 34509592 10.1016/j.jaac.2021.08.021 PMC8904650

[76] S. Stack, Suicide: A 15-year review of the sociological literature. Part I: Cultural and economic factors. Suicide Life Threat Behav. **30**, 145–162 (2000). 10888055

[77] A. Forte , Suicide risk among immigrants and ethnic minorities: A literature overview. Int. J. Environ. Res. Public Health **15**, 1438 (2018). 29986547 10.3390/ijerph15071438 PMC6068754

[78] T. E. Joiner, Why People Die by Suicide (Harvard University Press, Cambridge, MA, 2005).

[79] J. D. Ivanich , Social network differences between American Indian Youth who have attempted suicide and have suicide ideation. Community Ment. Health J. **58**, 589–594 (2022). 34196904 10.1007/s10597-021-00857-y PMC8929287

[80] A. Martínez-Hernáez, N. Carceller-Maicas, S. M. DiGiacomo, S. Ariste, Social support and gender differences in coping with depression among emerging adults: A mixed-methods study. Child Adolesc. Psychiatry Ment. Health **10**, 2 (2016). 26744601 10.1186/s13034-015-0088-x PMC4704269

[81] S. Hinshaw, R. Kranz, The Triple Bind: Saving Our Teenage Girls from Today’s Pressures (Ballantine Books, New York, 2009).

[82] N. Slesnick , Cognitive therapy for suicide prevention: A randomized pilot with suicidal youth experiencing homelessness. Cogn. Therapy Res. **44**, 402–411 (2020).

[83] M. Ferrada-Noli, M. Asberg, K. Ormstad, P. Nordström, Definite and undetermined forensic diagnoses of suicide among immigrants in Sweden. Acta Psychiatr. Scand. **91**, 130–135 (1995). 7778471 10.1111/j.1600-0447.1995.tb09753.x

[84] S. Stryker, P. J. Burke, The past, present, and future of an identity theory. Soc. Psychol. Q. **63**, 284–297 (2000).

[85] G. M. Zimmerman, C. Rees, C. Posick, L. A. Zimmerman, The power of (Mis)perception: Rethinking suicide contagion in youth friendship networks. Soc. Sci. Med. **157**, 31–38 (2016). 27060539 10.1016/j.socscimed.2016.03.046

[86] B. A. Pescosolido, “Bringing the “community” into utilization models: How social networks link individuals to changing systems of care” in Research in the Sociology of Health Care, J. Kronenfeld, Ed. (JAI Press, Greenwich, CT, 1996), **vol. 13**, pp. 171–198.

[87] S. Clegg, M. P. E. Cunha, A. Rego, Explaining suicide in organizations: Durkheim revisited. Bus. Soc. Rev. **121**, 391–414 (2016).

[88] S. Abrutyn, A. S. Mueller, M. Osborne, Rekeying cultural scripts for youth suicide: How social networks facilitate suicide diffusion and suicide clusters following exposure to suicide. Soc. Mental Health **10**, 112–135 (2019).

[89] S. H. Stephan, M. Weist, S. Kataoka, S. Adelsheim, C. Mills, Transformation of children’s mental health services: The role of school mental health. Psychiatr. Serv. **58**, 1330–1338 (2007). 17914011 10.1176/ps.2007.58.10.1330

[90] L. M. Menger, L. Stallones, J. E. Cross, K. L. Henry, P. Y. Chen, Strengthening suicide prevention networks: Interorganizational collaboration and tie strength. Psychosocial Intervention **24**, 155–165 (2015).

[91] A. K. Hagaman, U. Maharjan, B. A. Kohrt, Suicide surveillance and health systems in Nepal: A qualitative and social network analysis. Int. J. Mental Health Syst. **10**, 46 (2016). 10.1186/s13033-016-0073-7 PMC4895957 27274355

[92] C. R. Glenn , Annual research review: A meta-analytic review of worldwide suicide rates in adolescents. J. Child Psychol. Psychiatry **61**, 294–308 (2020). 31373003 10.1111/jcpp.13106

[93] J. A. Hoffmann, C. A. Farrell, M. C. Monuteaux, E. W. Fleegler, L. K. Lee, Association of pediatric suicide with county-level poverty in the United States, 2007–2016. JAMA Pediatr. **174**, 287–294 (2020). 31985759 10.1001/jamapediatrics.2019.5678 PMC6990805

[94] M. Oyesanya, J. Lopez-Morinigo, R. Dutta, Systematic review of suicide in economic recession. World J. Psychiatry **5**, 243–254 (2015). 26110126 10.5498/wjp.v5.i2.243 PMC4473496

[95] S. Stack, Contributing factors to suicide: Political, social, cultural and economic. Prev. Med. **152**, 106498 (2021). 34538366 10.1016/j.ypmed.2021.106498

[96] D. S. Chon, National religious affiliation and integrated model of homicide and suicide. Homicide Stud. **21**, 39–58 (2017).

[97] N. Zadravec Šedivy, T. Podlogar, D. C. R. Kerr, D. De Leo, Community social support as a protective factor against suicide: A gender-specific ecological study of 75 regions of 23 European countries. Health Place **48**, 40–46 (2017). 28934635 10.1016/j.healthplace.2017.09.004

[98] Z. P. Neal, The Connected City: How Networks Are Shaping the Modern Metropolis (Routledge, New York, ed. 1, 2012).

[99] Y. Guo , The geography of suicide in older adults in Hong Kong: An ecological study. Int. J. Geriatr. Psychiatry **35**, 99–112 (2020). 31663178 10.1002/gps.5225

[100] A. Sankar , Graph theory analysis of whole brain functional connectivity to assess disturbances associated with suicide attempts in bipolar disorder. Transl. Psychiatry **12**, 7 (2022). 35013103 10.1038/s41398-021-01767-z PMC8748935

[101] X. Liu , Alterations of core structural network connectome associated with suicidal ideation in major depressive disorder patients. Transl. Psychiatry **11**, 243 (2021). 33895787 10.1038/s41398-021-01353-3 PMC8068724

[102] A. Stumps , Connectome-based functional connectivity markers of suicide attempt. J. Affect. Disord. **283**, 430–440 (2021). 33549365 10.1016/j.jad.2020.11.061

[103] A. Jagger-Rickels , Aberrant connectivity in the right amygdala and right middle temporal gyrus before and after a suicide attempt: Examining markers of suicide risk. J. Affect. Disord. **335**, 24–35 (2023). 37086805 10.1016/j.jad.2023.04.061 PMC10330566

[104] C. R. Chin Fatt , Leveraging the microbiome to understand clinical heterogeneity in depression: Findings from the T-RAD study. Transl. Psychiatry **13**, 139 (2023). 37117195 10.1038/s41398-023-02416-3 PMC10147668

[105] I. Otsuka , Genome-wide association studies identify polygenic effects for completed suicide in the Japanese population. Neuropsychopharmacology **44**, 2119–2124 (2019). 31476763 10.1038/s41386-019-0506-5 PMC6887868

[106] J. S. Mattick, M. J. Gagen, The evolution of controlled multitasked gene networks: The role of introns and other noncoding RNAs in the development of complex organisms. Mol. Biol. Evol. **18**, 1611–1630 (2001). 11504843 10.1093/oxfordjournals.molbev.a003951

[107] J. Neira-Fresneda, L. Potocki, Neurodevelopmental disorders associated with abnormal gene dosage: Smith-Magenis and Potocki-Lupski syndromes. J. Pediatr. Genet. **4**, 159–167 (2015). 27617127 10.1055/s-0035-1564443 PMC4918721

[108] M. Shirani , Increased protein kinase A activity induces fibrolamellar hepatocellular carcinoma features independent of DNAJB1. Cancer Res., 10.1158/0008-5472.Can-23-4110 (2024). PMC11325150 38888469

[109] Rockefeller University, Rare liver cancer has surprising origins. Futurity https://www.futurity.org/rare-cancer-surprising-origins-3232962/. (24 June 2024).

[110] M. Sokolowski, J. Wasserman, D. Wasserman, Genome-wide association studies of suicidal behaviors: A review. Eur. Neuropsychopharmacol. **24**, 1567–1577 (2014). 25219938 10.1016/j.euroneuro.2014.08.006

[111] B. A. Pescosolido, B. Lee, K. Kafadar, Cross-level sociodemographic homogeneity alters individual risk for completed suicide. Proc. Natl. Acad. Sci. U.S.A. **117**, 26170–26175 (2020). 33020285 10.1073/pnas.2006333117 PMC7584914

[112] B. A. Pescosolido, The social context of religious integration and suicide: Pursuing the network explanation. Sociol. Q. **31**, 337–357 (1990).

[113] V. Ajdacic-Gross , Reduction in the suicide rate during advent–A time series analysis. Psychiatry Res. **157**, 139–146 (2008). 17976737 10.1016/j.psychres.2006.07.014

[114] V. Ajdacic-Gross, M. Bopp, M. Ring, F. Gutzwiller, W. Rossler, Seasonality in suicide–A review and search of new concepts for explaining the heterogeneous phenomena. Soc. Sci. Med. **71**, 657–666 (2010). 20573433 10.1016/j.socscimed.2010.05.030

[115] Y. Xiao, M. A. Lindsey, Adolescent social networks matter for suicidal trajectories: Disparities across race/ethnicity, sex, sexual identity, and socioeconomic status. Psychol. Med. **52**, 1–12 (2021). 10.1017/S0033291721000465 PMC9772914 33653436

[116] M. Skinner, N. Y. Miller, Aggregation and social interaction in garter snakes (Thamnophis sirtalis sirtalis). Behav. Ecol. Sociobiol. **74**, **51** (2020).

[117] P. Nicaise , Implementation of a computer-assisted face-to-face intervention for mapping the social support networks of patients with severe mental illness in routine clinical practice: Analysis of the appropriateness and acceptability of the intervention. Int. J. Soc. Psychiatry **68**, 1774–1782 (2022). 34791955 10.1177/00207640211058977

[118] P. Zhu , Correlated evolution of social organization and lifespan in mammals. Nat. Commun. **14**, 372 (2023). 36720880 10.1038/s41467-023-35869-7 PMC9889386

[119] C. E. Martin, G. A. Fox, B. J. Putman, W. K. Hayes, Social security: Can rattlesnakes reduce acute stress through social buffering? Front. Ethol. **2**, 1181774 (2023).

